# Vitronectin Absorbed on Nanoparticles Mediate Cell Viability/Proliferation and Uptake by 3T3 Swiss Albino Mouse Fibroblasts: In Vitro Study

**DOI:** 10.1155/2013/539348

**Published:** 2013-02-28

**Authors:** F. Rosso, G. Marino, A. Grimaldi, G. Cafiero, E. Chiellini, F. Chiellini, M. Barbarisi, A. Barbarisi

**Affiliations:** ^1^Laboratory of Applied Biotechnology, Department of Anaesthesiological, Surgical and Emergency Sciences, Second University of Napoli, Via Costantinopoli 16, 80138 Napoli, Italy; ^2^Laboratory of Bioactive Polymeric Materials for Biomedical and Environmental Applications, UdR INSTM, Department of Chemistry and Industrial Chemistry, University of Pisa, Via Risorgimento 35, 59126 Pisa, Italy

## Abstract

We study the interaction of 3T3 Swiss albino mouse fibroblasts with polymeric nanoparticles (NPs) and investigate cellular behaviour in terms of viability/cytotoxicity, cell cycle, NPs uptake, MAP kinase (ERK1/2), and focal adhesion kinase (FAK) activation. After incubation of NPs with cell culture media, western blot analysis showed that Vitronectin is retained by NPs, while Fibronectin is not detected. From cytotoxicity studies (MTT and BrdU methods) an LD50 of about 1.5 mg/mL results for NPs. However, NPs in the range 0.01–0.30 mg/mL are able to trigger a statistically significant increase in proliferation and cell cycle progression in dose and time depending manner. Also, biochemical evaluation of ERK1/2 and FAK clearly shows an increasing phosphorylation in a dose and time depending manner. Finally, we found by transmission electron microscopy that NPs are internalised by cells. Competitively blocking VN-integrin receptors with echistatin (1 *μ*g/mL) results in a decrease of viability/proliferation, cell cycle progression, cellular uptake, and FAK/ERK activation showing the involvement of Vitronectin receptors in signal transduction. In conclusion, our results show that cell surface NPs interactions are mediated by absorbed plasma proteins (i.e., Vitronectin) that represent an external stimuli, switched to the nucleus by FAK enzyme, which in turn modulate fibroblasts viability/proliferation.

## 1. Introduction

Many papers are present in literature concerning the nanoparticles (NPs) tailoring and their biological applications [1–3].

Increasing evidence indicates that in biological fluids proteins associate with NPs to form a “corona,” and that the amount and the structural/functional properties of the adsorbed proteins shape the interactions of these nanomaterials with the cells and potentially contribute to their biological responses [4–8].

Hence, NPs do not interact directly with the cells but is the protein coronas that play an essential role in the interaction with lipids or protein receptors of the cell membrane [9–11]. Recently, the structural characteristics of protein coronas and its relation with NPs surface coatings have been studied [6, 7]. These studies showed that when NPs are put in contact with plasma proteins, hard (stable) and soft (labile) protein layers are formed on NPs surface. It seems that stable linked proteins retain their native properties, while labile linked protein may undergo surface denaturation, loosing their native properties [7].

In addition, activation of cell signalling pathways by interaction of magnetic NPs with proteins on the cell surface is increasingly studied in particular for mechanosensitive cell receptors [12]. To this end, magnetic NPs are coated with specific ligands that enable them to bind specifically to receptors on the cell surface, as in the case of Fibronectin bounded NPs for integrin targeting [13]. Recent studies have clearly identified a number of serum proteins that bind to CB, TiO_2_, or acrylamide NPs [14–16]. Even though the structural and functional statuses of these proteins absorbed on the NP surfaces have not been extensively investigated, it is clear that they may contribute to the biological effects of NPs through activation/inactivation of receptor-dependent signalling [12, 17, 18], that in turn regulate important cell parameters such as viability/proliferation, differentiation, cell cycle, and cellular uptake.

Stimulation of cell surface by the external environment leads to the activation of kinase cascades that integrate and amplify extracellular signals and carry “messages” from the cell membrane to intracellular targets coordinating many complex biological functions [19].

Focal adhesion kinase (FAK) is a ubiquitously expressed nonreceptor protein tyrosine kinase that has emerged as a crucial molecule in integrating signals from integrins and receptor tyrosine kinases in processes such as cell survival, proliferation and motility [20, 21].

Ligand binding of integrins results in catalytic activation of FAK and in its autophosphorylation at tyrosine residue in position 397 (Y397), which serves as a binding site for several Src homology 2 (SH2) domain-containing proteins, including Src kinases [22].

The FAK/Src dual kinase complex leads to further phosphorylation of FAK and also to phosphorylation and activation of a number of cytoskeleton-linked proteins, which transduce signals to downstream pathways, such as the mitogen-activated protein (MAP) kinase cascades that control cell survival, motility, and proliferation [23, 24]. 

At present, the molecular mechanisms that control FAK are still under investigation.

However, the first event that happens as consequence of cell interaction with external stimuli is represented by the modulation of membrane receptors, such as integrins [20].

Integrins are heterodimeric transmembrane receptors composed of eighteen *α* subunits and eight *β* subunits that can be noncovalently assembled into 24 combinations. Therefore, the specific integrin expression patterns by a cell dictate which extra cellular matrix (ECM) substrate that the cell can bind [25], and the composition of integrin adhesomes, a protein complex bound to the cytoplasmic portion of integrin receptors, determines the downstream signalling events, thus the eventual cell behaviour and fate. Integrins have unique ability to respond to the molecular composition and physical properties of the ECM and integrate both mechanical and chemical signals through direct association with the cytoskeleton, which also determines the selection of specific integrin species to be involved. Integrin recognises and binds to the Arg-Gly-Asp (RGD) motif which was first discovered in Fibronectin, but later found in many other ECM proteins including Vitronectin [26].

Fibronectin and Vitronectin are two major cell-adhesive proteins in mammalian sera. Past studies, demonstrated that cell adhesion and spreading correlate with adsorption of Fibronectin and/or Vitronectin to the culture substratum from serum-containing medium suggested that these proteins accounted for most of the adhesive/cell spreading activity in serum [27, 28].

At the aim to study the interaction between cell and nanoobjects, we choose a NPs system, tailored for the delivery of antifibrinolitic drugs, prepared by applying the *coprecipitation* technique, and based on alternating copolymers of maleic anhydride with alkyl vinyl ethers containing 1-*O*-glycidyl-2,3-*O*-isopropylidenglycerol-*β*-cyclodextrin as formulation stabilizer [29–32]. Firstly we studied the NPs effects on cell viability/proliferation, cell cycle progression, and cellular uptake, and then we verified, in vitro, the interaction with Fibronectin and Vitronectin, two major cell-adhesive proteins of mammalian sera.

Hence, this paper represent a further contribute to the elucidation of fine biochemical pathway triggered by nanoparticle interaction with cell systems.

## 2. Materials and Methods

### 2.1. Cell Culture

Stock cultures of Swiss 3T3 cells were maintained in Dulbecco's modified Eagle's medium (DMEM), supplemented with 10% fetal bovine serum (FBS) in humidified 5% CO_2_, 95% air atmosphere at 37°C. For experimental purposes, Swiss 3T3 cells were plated in 100 mm dishes at 6 × 10^5^ cells/dish in DMEM containing 10% fetal bovine serum and used after 1-2 days when the cells were half-confluent (about 50–60%). 

### 2.2. Nanoparticles Preparation and Characterisation

Nanoparticles were prepared by applying the *coprecipitation* technique to an alternating copolymers of maleic anhydride with alkyl vinyl ethers (VAM41 polymer) as reported elsewhere [29–32]. Briefly, 50 mg of polymer were dissolved in 2 mL of 4 : 1 ethanol/water solutions, added drop wise (22G needle) to 5 mL of deionised water, containing 125 mg of 1-*O*-glycidyl-2,3-*O*-isopropylidenglycerol-*β*-cyclodextrin (GIG-*β*CD) and 20 mg of human serum albumin (HSA), and kept under magnetic stirring. The dropping rate was adjusted to 30 drops/minute, and the process took place at room temperature.

Nanoparticle suspensions were purified by centrifugation in ALC PK121R centrifuge at 8000 g for 30 minutes at 4°C, and the pellets were suspended in appropriate medium for subsequent experiments. 

Dimensional analyses that were carried out by Coulter LS230 Laser Diffraction Particle Size Analyzer equipped with *small volume module plus.* Diameter distribution was processed using Fraunhofer optical model. Three runs were performed on each sample.

Zeta potential analyses were carried out by using a Coulter Beckman Delsa 440SX at 25°C with a 0.4°C tolerance between upper and lower sensors. Nanoparticle suspensions were purified, redispersed in 0.9% NaCl solution, pH 5–5.2, and diluted to a final concentration of 0.1 mg/mL. Zeta potential values were calculated as the mean value of 10 replicates for each nanoparticle formulation.

### 2.3. Cell Proliferation Assay

Cell viability and proliferation was evaluated by mitochondrial dehydrogenase assay (MTT) and Bromouridine (BrdU) incorporation methods.

Briefly, quantification of mitochondrial dehydrogenase activity was achieved via the enzymatic conversion of MTT tetrazolium water soluble salt to a coloured formazan product. Since reduction of MTT occurs only in metabolically active cells, the level of activity was a measure of cell viability. 

For experimental purpose fibroblasts were seeded in a 24-well plate at a density of 1 × 10^4^ cells/well in 1 mL of complete medium for 24 h, after which the growth medium was removed and replaced with the medium containing nanoparticles. For control experiments, medium without NPs was used.

Fibroblasts were cultured with NPs at concentration of 0.01, 0.03, 0.10, 0.30, 0.60, 1, and 1.5 mg/mL for 0.5, 2, 6, and 24 h (contact time) at 37°C in 5% CO_2_. After, cells were washed in PBS three times to remove the NPs excess, fresh medium containing MTT soluble salt was added according to manufacturer instructions.

Fibroblast viability was calculated as O.D._450 nm_ percentage with respect to control cells. Statistical data analysis was achieved calculating media and standard deviation, and each experimental point was performed in quintuplicate.

BrdU incorporation method was performed according to manufacturer recommendation (http://www.roche-applied-science.com/) with some modifications. Briefly, fibroblasts at 50% confluence were incubated with growth medium containing NPs at 0.01, 0.03, 0.10, 0.30, 0.60, 1.0, and 1.5 mg/mL for 3, 6, and 24 hours in incubator at 37°C and 5% CO_2_. For experiments at 6 and 24 hours, 10 *μ*M BrdU were added in the culture medium 3 hours before the end of incubation time. For the experiments at 3 hours, 10 *μ*M BrdU were added in the culture medium together with nanoparticles. In this way, cells were held in contact with nanoparticles for 3, 6, and 24 hours and every 3 hours with BrdU. After washed twice in PBS, labelled cells were trypsinized, washed in PBS, and fixed in a 70 : 30 vol/vol 50 mM glycine water solution in ethanol (pH adjusted at 2.0 with HCl) for 30 min at 4°C. After washed twice in PBS, cell pellets were then incubated in 4N HCL for 15 min and washed in PBS.

Cells were then resuspended in PBS containing 0.1% Tween 20 and 0.5% BSA for 10 min. After centrifugation pellets were resuspended in a 1 : 20 dilution in PBS containing 0.1% Tween 20 and 0.5% BSA of anti-BrdU fluorescein conjugated antibody for 45 min at 37°C. 

Cells were again washed twice in PBS then resuspended in PBS and analyzed on a Becton Dickinson FACScan analyzer to quantify fluorescein fluorescence (excitation wavelength *λ*_ex_ = 488 nm and emission wavelength *λ*_em_ = 515 nm) at the single cell level. Data were analyzed using Cellquest1version 3.3 software. In total, 6,000 events were acquired, but noncellular particles and debris (located on the bottom left corner of the dot plot) were excluded by prior gating, thereby limiting undesired effects on overall fluorescence. Final gated cell populations usually contained 5,000 cells. Each experimental point was performed in triplicate.

At the aim to evaluate if Vitronectin receptors are involved in cell viability, experiments (MTT and BrdU) were performed also in presence of 1 *μ*g/mL of echistatin. Echistatin is a natural disintegrin found in snake venom and binds irreversibly to a_v_b_3_ integrin, mimicking Vitronectin [33, 34].

### 2.4. Cell Cycle Analysis

To evaluate the effect of NPs on cell cycle, fibroblasts were cultured overnight in 25 cm^2^ flasks at a density of 4 × 10^3^ cells/cm^2^ in 5 mL of medium without FBS (synchronization). The day after the growth medium was removed and replaced with complete medium containing nanoparticles at concentration of 0.01, 0.03, 0.10, and 0.30 mg/mL. At the aim to evaluate if Vitronectin receptors are involved in cell cycle regulation, experiments at 0.30 mg/mL were performed also in presence of 1 *μ*g/mL echistatin. In control experiments, complete medium without particles was used.

Cells belonging to both experimental sections were harvested and processed by flow cytometry.

About 1 × 10^6^ cells/tube were washed with ice-cold PBS and fixed in 75% ethanol at 4°C overnight. Fixed cells were washed twice with PBS and treated with 10 *μ*g/mL DNase-free RNase at 37°C for 30 min. DNA was stained using 10 *μ*g/mL PI at 37°C for 30 min in the dark.

Samples were analyzed using a flow cytometer (FACSCalibur TM, Becton Dickinson, San Jose, CA) with excitation at 488 nm and emission at 530 ± 30 nm through a DF 530/30 filter. In all experiments, 10,000 events were recorded. The data were analyzed using Cellquest software (Becton Dickinson, San Jose, CA). The proportion of cells in the G0/G1, S, and G2/M phases was determined.

### 2.5. Immunoprecipitation

Swiss 3T3 fibroblasts were seeded in 100 mm dishes at density of 1 × 10^4^ cells/cm^2^ in DMEM containing 10% fetal bovine serum. After 1-2 days, when the cells were at 60–70% of confluence, the growth medium was removed and replaced with medium containing NPs at 0.01, 0.03, 0.10, and 0.30 mg/mL for a selected time intervals; experiments at 0.30 mg/mL were performed also in presence of 1 *μ*g/mL of echistatin. Control experiments were performed in medium without NPs.

At selected time intervals cells were harvested with a scraper in ice and lysated in 0.2 mL of ice-cold RIPA buffer with 50 mM HEPES, pH 7.4, 1% Triton X-100, 1% sodium deoxycholate, 0.1% SDS, 150 mM NaCl, 10% glycerol, 1.5 mM MgCl_2_, 1 mM EGTA, 1 mM sodium orthovanadate, 10 mM sodium pyrophosphate, 100 mM NaF, and 1 mM phenylmethylsulfonyl fluoride and protease inhibitor cocktail from SIGMA as suggested by the supplier.

Lysates were clarified by centrifugation at 15,000 g for 10 min, and the pellets discarded. After centrifugation, supernatants were transferred to fresh tubes then to protein assay (Bio-Rad). Successively, samples were normalized for protein concentration, were divided in two aliquots, and immunoprecipitated (4°C, 2 h), respectively, with protein-A Sepharose linked to polyclonal anti-ERK1/2 antibody and polyclonal anti-FAK antibody [35]. Immunoprecipitates were rinsed three times with RIPA buffer, extracted in 2X SDS-PAGE sample buffer (200 mM Tris-HCl, pH 6.8, 1 mM EDTA, 6% SDS, 4% 2-mercaptoethanol, 10% glycerol), by boiling 5 min, and resolved by 10 to 20% polyacrylamide linear gradient (ERK1/2) and 7% SDS-PAGE (FAK).

### 2.6. Western Blotting

After SDS-PAGE, proteins were transferred to polyvinylidene difluoride (PVDF) membranes (one for ERK1/2 and the other for FAK). 

Membranes were blocked for 1 h with 5% nonfat dried milk in Tris buffer saline + Tween 20 0.05% pH 7.2 (TBST) and incubated for 2.5 h at 4°C with antiphospho-ERK1/2 monoclonal antibody (0.1 mg/mL) or antiphospho-FAK(tyr 397) monoclonal antibody (0.1 mg/mL), respectively. The membranes were rinsed three times with TBS-0.05% Tween 20, incubated with secondary antibodies (horseradish peroxidase-conjugated goat antirabbit IgG antibody), and diluted 1 : 5,000 in Phosphate Buffer solution (PBS), for 1 h at room temperature. After rinsing three times with TBS Tween 20 0.05%, the immunoreactive bands were visualized with enhanced chemiluminescence detection reagents (ECL, Bio-Rad Hercules CA). Protein signals on PVDF membranes were assessed with the ChemiDoc XRS imaging densitometer (Bio-Rad, US), using the Quantity One software program (Bio-Rad, USA).

For detecting total ERK1/2 and FAK signals, PVDF membranes were stripped with 0.25 M Tris-HCl pH 6.8, 2% SDS and 100 mM *β*-Mercaptoethanol and reprobed with with anti-ERK1/2 or anti-FAK antibody (incubated overnight at 4°C). Visualization and quantification were performed with the same procedure reported above for phosphokinases.

ERK 1/2 and FAK activations per sample were calculated using the following formula: phosphoKinase/Total Kinase × 100, where phosphokinase was the densitometer signals intensity of phosph-ERK or phospho-FAK and, Total Kinase was the densitometer signal intensity of total ERK or total FAK as described by Rosso et al. [36].

### 2.7. TEM Analysis

3T3 Swiss albino mouse fibroblasts were cultured in 100 mm dishes at 6 × 10^5^ cells/dish in DMEM containing 10% fetal bovine serum at 37°C in 5% CO_2_. After 1-2 days, when cells reach 60–70% of confluence, the growth medium was removed and replaced with the medium containing NPs (0.03 mg/mL and 0.30 mg/mL).

For control experiments, medium without nanoparticles was used. After 24 hours of culture, the cells were fixed in 3% glutaraldehyde in 0.065 M phosphate buffer (pH 7.4) for 2 hr at room temperature. The specimens were then postfixed into 1% OsO4 in 0.1 M phosphate buffer (pH 7.4) at 4°C before being dehydrated with ethanol and acetone and embedded in Spurr's resin.

Thin sections, obtained with a diamond knife on a Supernova ultramicrotome, were sequentially stained at room temperature with 2% uranyl acetate (aqueous) for 5 min and by lead citrate for 10 min. Ultrastructural observations were made using a FEI EM 208 transmission electron microscope (TEM) in SYS MegaVIEW II digital mode.

### 2.8. Vitronectin and Fibronectin Detection

To determine the amount of Vitronectin and Fibronectin that interact with our NPs, they were incubated at 0.30 mg/mL with 3T3 fibroblast culture media in humidified incubator 5% CO_2_, 95% air atmosphere at 37°C. After 1 hour, tubes were centrifuged at 8000 g for 30 minutes at 4°C, supernatants were removed and NPs samples were retrieved and washed three times with PBS to remove loosely bound proteins. Third washing did not contained proteins as demonstrated by Bradford protein assay (Bio-Rad, Milan, Italy).

Proteins remaining on the NPs were solubilized in boiling sodium dodecyl sulfate (SDS) buffer (50 mM Tris, 2% SDS, 5% *β*-mercaptoethanol) for 30 minutes, with constant agitation. The supernatants were collected and resolved on a 7% polyacrylamide gel.

After SDS-PAGE, proteins were transferred to polyvinylidene difluoride (PVDF) membranes.

Membranes were blocked for 1 h with 5% nonfat dried milk in Tris buffer saline + Tween 20 0.05% pH 7.2 (TBST) and incubated for 2.5 h at 4°C with antiFibronectin monoclonal antibody (Chemicon-Millipore, Italy; 0.1 mg/mL) or anti-Vitronectin monoclonal antibody (Santa Cruz, California USA; 0.1 mg/mL). The membranes were rinsed three times with TBS 0.05% Tween 20, incubated with secondary antibodies (horseradish peroxidase conjugated goat antirabbit IgG antibody), and diluted 1 : 5,000 in Phosphate Buffer solution (PBS) for 1 h at room temperature. After rinsing three times with TBS 0.05% Tween 20, the immunoreactive bands were visualized with enhanced chemiluminescence detection reagents (ECL, Bio-Rad Hercules CA). Protein signals on PVDF membranes were assessed with the ChemiDoc XRS imaging densitometer (Bio-Rad, USA), using the Quantity One software program (Bio-Rad, USA).

## 3. Results

### 3.1. Nanoparticles Preparation and Characterisation

Homogeneous nanoparticles suspensions were characterised from a chemical-physical point of view, performing granulometry in suspension and Zeta potential measurements. As reported elsewhere [29–32], NPs displayed a mean diameter of (137 ± 22) nm and a negative surface charge (−18.5 ± 1.1 mV), generated by the carboxyl ions belonging to the chain of the polymers. 

### 3.2. Proliferation Assays

**Figure 1:**
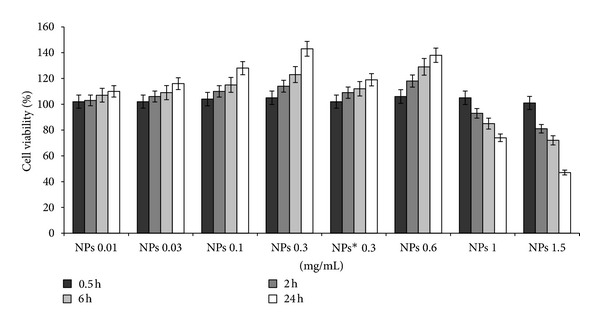
Fibroblasts viability/proliferation by MTT method. 3T3 Swiss albino mouse fibroblasts in contact with NPs at indicated time and concentrations.

**Figure 2:**
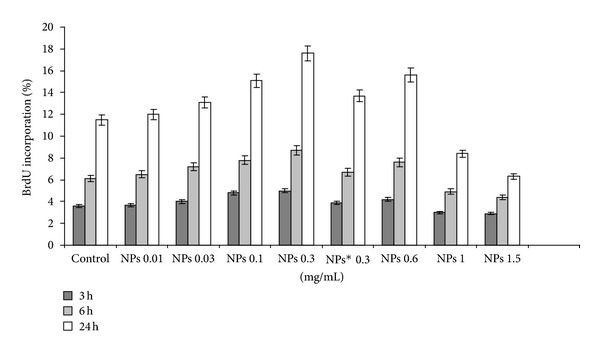
Fibroblasts incubated with NPs, viability/proliferation by BrdU percentage incorporation method.

As shown in Figure 1, NPs in the concentration range 0.01–0.3 mg/mL increased fibroblasts viability in a dose and time depending manner, while at higher concentration a decrease in cell viability was evident, resulting in an IC_50_ of about 1.5 mg/mL. Hence, 0.3 mg/mL was suitable as upper concentration limit for the subsequent NPs studies. These data were not surprising if we considered that alternating copolymers of maleic anhydride with alkyl vinyl ethers show a IC_50_ in the range 0.5–0.7 mg/mL [30]. Substantially, also BrdU cell proliferation experiments (Figure 2) were similar with MTT assay, confirming an increase in cell number for fibroblast incubated with NPs at low concentration. Interestingly, when experiments were performed in presence of 1 *μ*g/mL of echistatin (inhibitor of integrin), both methods showed a decrease of cell viability/proliferation (Figures 1 and 2).

### 3.3. Cell Cycle Analysis

**Figure 3:**
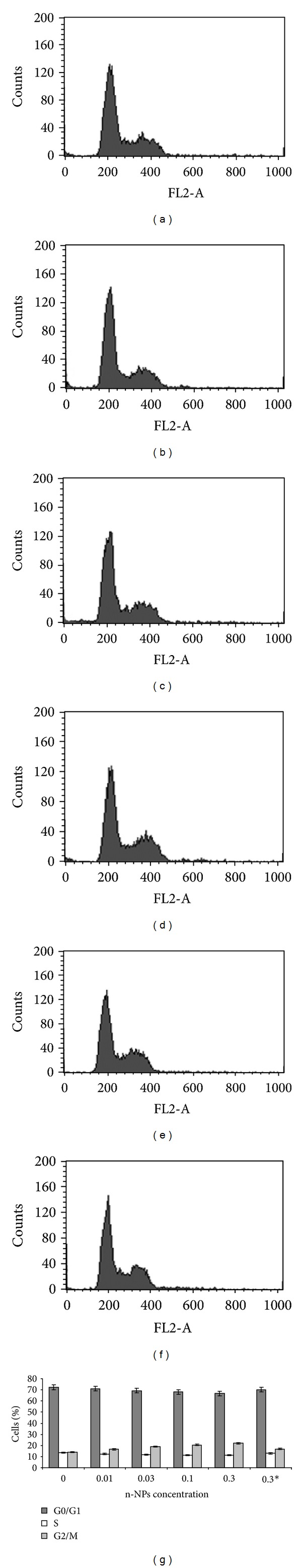
Cell cycle analysis. (a) Fibroblasts without NPs; (b), (c), (d), and (e) cells incubated with NPs 0.01, 0.03, 0.10, and 0.30 mg/mL, respectively; (f) cells incubated with NPs 0.30 mg/mL plus 1 *μ*g/mL echistatin; (g) Graph illustrating cells distribution in G0/G1, S, and G2/M phases. 0.3* indicate samples treated with echistatin.

The effect of NPs on cell cycle is shown in Figure 3. As we can see, NPs slightly enhance the percentage of fibroblasts in G2/M phase in a time and dose depending manner.

However, in presence of echistatin (1 *μ*g/mL), we found that cells were able to return to a physiological behaviour; in fact, percentages of cells in G0/G1, S, and G2/M were comparable with control fibroblasts. 

### 3.4. ERK1/2 Activation

Mitogen-activated protein kinases (MAPKs) are involved in regulation of cellular responses leading to cell growth, differentiation, cell cycle progression, and apoptosis in mammalian cells. In general, extracellular regulated kinases 1/2 (ERK1/2) cascade is a critical pathway for mitogenesis and differentiation. It is reasonable to postulate that NPs induced cell proliferation could be triggered, at least in part, through ERK1/2 modulation.

**Figure 4:**
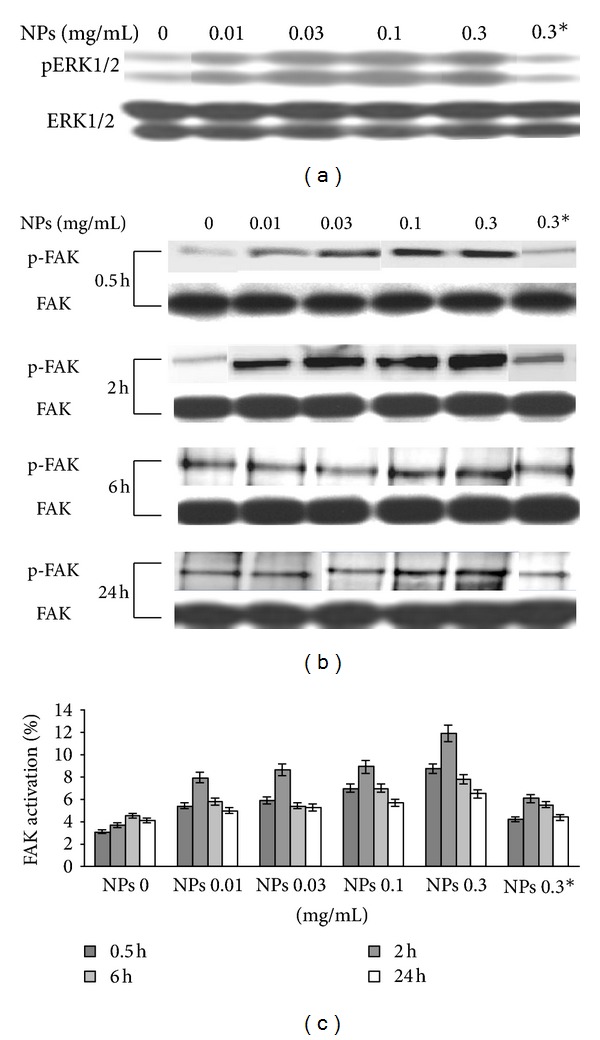
ERK1/2 and FAK activation. (a) PosphoERK1/2 and total ERK1/2 western blot signals; (b) posphoFAK and total FAK western blot signals; (c) graph illustrating the densitometric analysis of the ratio phosphoFAK (tyr397) versus total FAK as *X* ± SD (*n* = 3).

To test this idea, 3T3 Swiss albino mouse cells were challenged with indicated NPs concentrations in standard medium. We found that signals of phospho-ERK1/2 increase after 24 hours in a dose depending manner, whereas the total ERK1/2 protein levels remained unchanged (Figure 4(a)). In agreement with cell proliferation results, dose-dependent activation of ERK1/2 signaling pathway was postulated to contribute in NPs-facilitated cell proliferation.

Interestingly, when experiments are performed in presence of 1 *μ*g/mL of echistatin, phospho-ERK1/2 signals are comparable with untreated cells (control).

### 3.5. FAK Activation

To test the hypothesis that cell proliferation via ERK1/2 activation is correlated to membrane NPs interaction, we evaluated FAK activation by western blot. 

The addition of NPs to fibroblasts culture medium induced an enhancement of FAK activation in early times (0.5 and 2 hours) and in dose depending manner (Figures 4(b) and 4(c)).

At higher contact times, the activation of FAK shows a decreasing trend that, however, never reaches levels of untreated cells. In presence of echistatin 1 *μ*g/mL, values of p397FAK are comparable with untreated fibroblasts. 

### 3.6. TEM Analysis

Transmission electron microscopy (TEM) was used for the study of NPs internalization by 3T3 Swiss albino mouse fibroblasts.

Experiments with NPs at low concentrations (0.03 mg/mL) were performed, but no internalization by fibroblasts after 24 hours was detected (data not showed).

**Figure 5:**
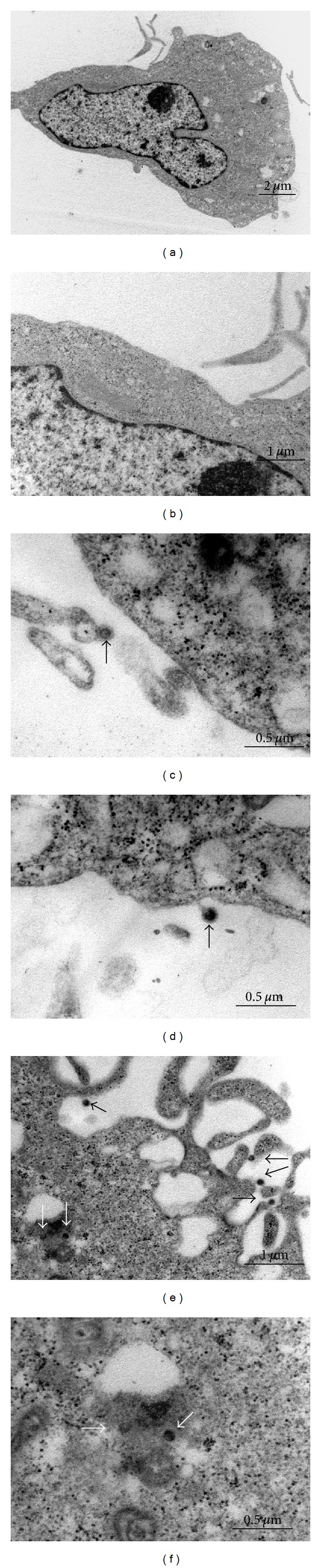
TEM micrographs of NPs uptake. (a) and (b) nontreated cells (controls); (c) and (d) fibroblasts treated with NPs 0.3 mg/mL and echistatin 1 *μ*g/mL; (e) and (f) fibroblasts in contact with NPs 0.3 mg/mL. Black arrows: extracellular NPs; white arrows: intracellular NPs.

Figure 5 shows TEM pictures of fibroblasts cultured in presence of NPs at 0.30 mg/mL and 24 hours of incubation. Pictures of control cells, without NPs, are also shown (Figures 5(a) and 5(b)). 

When fibroblasts are cultured, simultaneously, with NPs and echistatin 1 *μ*g/mL (Figures 5(c) and 5(d)), NPs are present only externally to cells.

As shown in Figures 5(e) and 5(f), NPs are internalised by fibroblasts after 24 hours of incubation time.

### 3.7. Fibronectin and Vitronectin Absorption on NPs

The amount of Vitronectin and/or Fibronectin absorbed on our NPs system were determined by western blot analysis. Vitronectin was detected on NPs samples, while Fibronectin was not detected (Figure 1). Vitronectin is a 75 KDa protein, that undergo chemical and enzymatic cleavage originating a 65 KDa and 10 KDa fragments [37]. Since we used highly denaturating conditions it is not surprising that we detect Vitronectin signal as two distinct bands (75–65 and 10 KDa). Preliminary experiments (western blot) indicate the absence of Fibronectin in our culture media; vice versa Vitronectin is detected (data not showed).

Probably, as reported in the literature [38], a loss of Fibronectin during industrial preparation of serum for cell culture is happened.

## 4. Discussion

There are many papers concerning the NPs interactions with various cell systems [12, 18, 19, 39]. In general, it is widely accepted that the surface of NPs plays an important and critical role in serum proteins adhesion [6–8] followed by linking with specific cell membrane receptors (i.e., integrins, etc.) inducing various biological responses.

The presence of *β*-cyclodextrin prevents proteins aggregation, thermal denaturation, and degradation [40, 41], so we assumed that *β*-cyclodextrin can minimize the vitronectin's denaturation when the NPs surface interacts with serum proteins. 

Our observations fetched the similar results of cell viability/proliferation enhancement with the interactions with NPs, that is also suggested in the literature [19, 42, 43] (Figures 1 and 2). However, NPs-cell interactions can depicted the elucidation of fine biochemical mechanisms triggered by NPs that attract attention to understand the cell response to external stimuli [12, 18, 19]. Hung et al. used polyurethane-gold nanocomposites to study cell behavior on nanophase-segregated materials, and their results suggested that the integrin *α*5*β*3/FAK pathway activation may be induced by nanophase-separated materials in both endothelial cell and fibroblasts to promote their proliferation/migration [43]. 

The aim of this study was to investigate in vitro behaviour of our NPs system with 3T3 fibroblast derived from Swiss albino mouse, that is tailored for the delivery of antifibrinolitic drugs. NPs interaction with fibroblasts having concentration and time dependent effect on viability/proliferation and cell cycle progression (Figures 1, 2, and 3). According to the MTT and BrdU assays, the improvement in viability of 3T3 fibroblast was observed because of induction of FAK and ERK1/2 kinases activation meanwhile echistatin showed the inhibitory effect. Its noteworthy to mention here that ERK1/2 belong to the family of mitogen-activated protein (MAP) kinases, that regulate cell survival, motility, and proliferation and were activated also by “external stimuli” at cell membrane [21, 22, 24], we speculated that NPs can induced cell proliferation, partly, through ERK1/2 modulation.

To evaluate NPs induced cell proliferation western blot analysis has been carried out, in which 3T3 Swiss albino mouse cells were exposed to different NPs concentrations in standard medium and determined the FAK and ERK1/2 protein band densities after specified time intervals. We found that signals of phosphorylated ERK1/2 enhanced in a dose depending manner, whereas the total ERK1/2 protein levels unchanged (Figure 4(a)).

In a similar western blot experiment, we found that signals of phosphorylated FAK kinase, that is also responsible for upregulation of ERK, enhance in a dose and time depending manner. 

**Figure 6:**
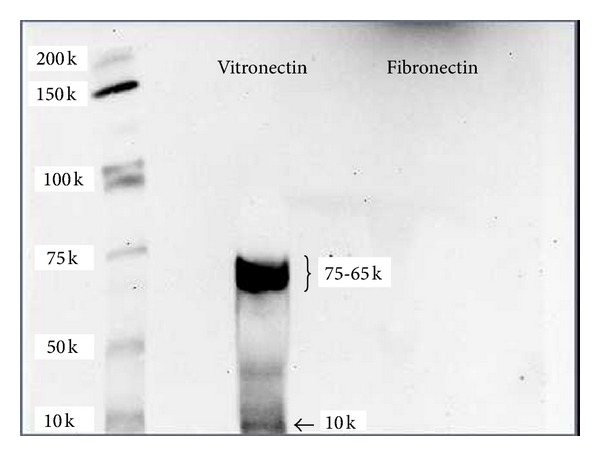
Western blot analysis of Fibronectin and Vitronectin adsorbed on NPs.

Since, we observed both ERK and FAK activation (Figure 4), it was reasonable to speculate that some serum proteins interact with our NPs system and triggered FAK/ERK proliferation pathway activation through integrins. In facts, western blot experiments showed that, Vitronectin interacts with our NPs system (Figure 6), while experiments with echistatin, clearly underlined the role of a_v_b_3_ integrin in the NPs modulation of cell viability and uptake (Figure 5).

Biochemical experiments with echistatin (Figure 4) clearly confirmed the role of both kinases in cell proliferation induced by contact with NPs; the addition of the disintegrin in culture media decreased both the phosphorylation of FAK and ERK1/2, bringing back the values at levels comparable with control cells (without NPs).

Using echistatin as integrin inhibitor, cell cycle experiments (Figure 3) showed an inhibition of NPs-induced cell cycle enhancement, confirming the dependence of cell cycle progression by NPs-a_v_b_3_ integrin interactions.

Our data, also showed that cellular uptake is dependent on NPs/a_v_b_3_ integrin interactions, since in presence of echistatin NPs is detected only externally to cells (Figure 5).

These experimental observations suggest the intriguing hypothesis that FAK would be involved also in NPs internalization, as in the case of virus uptake by cells [44].

However, further study are necessary to elucidate the role played by FAK and other kinases in the activation of specific cellular biochemical pathways triggered by NPs interaction with cellular systems.

## 5. Conclusions

Recently SDS-PAGE, Western Blot, mass spettroscopy, protein microarrays, dynamic light scattering, nanoparticle tracking analysis, differential centrifugal sedimentation, and infrared spectroscopy have been used to study the interaction between nanomaterials and the protein corona [6, 7, 45]. Using this forefront methodology has been demonstrated that biological effects depend by formation/composition of protein corona and NPs concentration/number in biological fluids.

The application of this methodology to our NPs system may explain some intriguing biological phenomena, such as the increase and the reduction of cell viability at low and high NPs doses, respectively. Possibly these studies (6,7) can evaluated Vitronectin's conformation, when it will interact with NPs that also depend on surface and concentration of nanoparticles.

In conclusion, our experimental data confirm the idea that the NPs protein corona triggers cellular response.
